# Optimized high-throughput whole-genome sequencing workflow for surveillance of influenza A virus

**DOI:** 10.1186/s13073-025-01512-x

**Published:** 2025-09-26

**Authors:** Matthias Licheri, Mike Mwanga, Manon F. Licheri, Annika Graaf-Rau, Cora Sägesser, Pascal Bittel, Timm Harder, Franziska Suter-Riniker, Jenna N. Kelly, Ronald Dijkman

**Affiliations:** 1 Institute for Infectious Diseases, University of Bern, Friedbühlstrasse 25, Bern, CH-3001 Switzerland; 2 Graduate School for Cellular and Biomedical Sciences, University of Bern, Bern, Switzerland; 3 Multidisciplinary Center for Infectious Diseases, University of Bern, Bern, Switzerland; 4 Institute of Virology and Immunology IVI, Bern and Mittelhäusern, Switzerland; 5 Department of Infectious Diseases and Pathobiology, Vetsuisse Faculty, University of Bern, Bern, Switzerland; 6 Institute of Diagnostic Virology, Friedrich-Loeffler-Institut, Greifswald-Insel Riems, Germany; 7 Department of Pathogen Evolution, Helmholtz Institute for One Health, Greifswald, Germany; 8 European Virus Bioinformatics Center, Jena, Germany

**Keywords:** Influenza A virus, Whole-genome sequencing, Oxford Nanopore sequencing, Swine IAV, Avian IAV, Human IAV, High-throughput

## Abstract

Whole-genome sequencing (WGS) is essential for monitoring the genetic diversity of influenza A virus (IAV) across host species. We optimized a multisegment RT-PCR (mRT-PCR) protocol to enhance amplification of all eight IAV segments using modified RT and PCR conditions. Additionally, we introduced a dual-barcoding approach for the Oxford Nanopore platform, enabling high-throughput multiplexing without compromising sensitivity. The resulting workflow is robust, scalable, and effective for avian, swine, and human IAV samples, even at low viral loads. This approach strengthens genomic surveillance at the human–animal interface, supporting early detection, evolutionary monitoring, and rapid identification of IAV spillover events.

**Supplementary Information:**

The online version contains supplementary material available at 10.1186/s13073-025-01512-x.

## Background

Influenza A viruses (IAVs), classified under the *Alphainfluenzavirus* genus and Orthomyxoviridae family, are negative-sense, single-stranded RNA viruses with a segmented genome of approximately 13.6 kb in size, encoding up to 17 proteins [1]. These viruses share antigenically related nucleocapsid and matrix proteins, but are classified based on their two surface glycoproteins, haemagglutinin (HA), with 19 recognized subtypes, and neuraminidase (NA), with 11 recognized subtypes [2, 3]. IAVs can be shed from their main reservoir, wild aquatic wild birds, to a wide range of avian and mammalian species, including pigs and humans. This transmission can lead to severe consequences for both animal and human health, as evidenced by the ongoing H5N1 panzootic, the increasing number of sporadic zoonotic spillovers, and the multiple human influenza pandemics that have occurred throughout history [2–9].

It is well established that point mutations caused by the error-prone viral polymerase or reassortment events resulting from simultaneous infection with two or more viral subtypes in the same host drive the genetic diversity and evolution of IAVs [10]. This genetic diversity can influence viral fitness, antiviral resistance, and interspecies transmissibility and is often associated with specific changes in one or more IAV genes or with the overall “constellation” of the genome segments. Therefore, whole-genome sequencing plays a pivotal role in monitoring IAV evolution in both humans and animals, enabling the detection of new variants and/or transmission patterns and assessing the efficacy of current vaccines and antiviral treatments. In addition, the availability of whole-genome sequences enables assessments of virulence, tropism, and zoonotic propensity. The one-step multisegment RT-PCR (mRT-PCR) approach pioneered by Zhou et al., or derivatives thereof, is often used for genomic surveillance of IAVs at the human–animal interface [11–18]. However, the recovery of sequence information for the genomic segments encoding the largest IAV genes, i.e., polymerase (PB1, PB2, and PA), can be quite challenging especially from clinical material with a low viral load. This problem is further pronounced when using third-generation sequencing platforms, which offer rapid and portable real-time long-read sequencing, but generally have a lower throughput than second-generation sequencing technologies [15].

To improve the sensitivity of recovering whole-genome sequences from avian, swine, and human IAV-positive clinical samples, we further optimized the existing approach from Rambo-Martin et al. [15] by using a different reverse transcription (RT) enzyme and adapting the RT and PCR cycling conditions. We then compared the performance of this optimized approach to previously established protocols by Zhou et al. and Rambo-Martin et al. [11, 12, 15]. This comparison revealed that optimization of individual RT and PCR reaction conditions improved the overall recovery of all eight genomic segments. Following these results, we also developed and tested a novel dual-barcoding approach to increase the sequencing throughput on portable third-generation sequencing platforms. This approach allowed multiplexing of at least eight IAV-positive samples of avian, swine, or human origin per sequencing library barcode without a significant loss in sensitivity, creating an optimized workflow for portable high-throughput whole-genome surveillance of influenza A viruses at the human–animal interface.

## Methods

### Clinical samples

In this study, we tested 24 IAV-positive clinical samples from human, swine, and avian hosts (8 samples per host). Human IAV-positive clinical samples were collected (nasal swabs) by physicians during routine clinical care in Bern, Switzerland, and submitted to the clinical diagnostic laboratory at the IFIK for routine viral diagnostics. All human samples were anonymous and had no identifiers. Sampling and sequencing were conducted in accordance with national regulations, and ethical approval was waived by the Cantonal Ethics Commission Bern (Req-2020–00167). Avian and swine IAV-positive clinical samples were originally collected and sequenced at the Friedrich-Loeffler-Institut (FLI) in Riems, Germany, as part of their routine molecular diagnostics for IAV. Extracted viral RNA (see below for protocol) was obtained from the FLI for each avian (combined swab) and swine (nasal or oropharyngeal swab) sample. Additional details for these 24 samples, including accession numbers, are summarized in Table S1.

### Viral RNA

To evaluate our IAV whole-genome amplification protocol in comparison to previously established methods [11, 12, 15], we prepared tenfold serial dilutions of A(H1N1)pdm09 virus stock in infection medium (iMEM) [19]. From each Dilution, 200 µL was used for viral RNA extraction using the KingFisher Apex automated platform (ThermoFisher Scientific, Waltham, MA, USA) in combination with the NucleoMag® VET kit (Macherey–Nagel, Düren, DE) following the manufacturer’s instructions. For human IAV-positive clinical samples, viral RNA was extracted from 200 µL of previously screened samples submitted for routine viral diagnostics by the treating physicians, in accordance with national regulations (Cantonal Ethics Commission Bern, Req-2020–00167) using the same extraction protocol. The viral RNA from avian and swine IAV-positive clinical samples was extracted previously as part of routine molecular diagnostics of IAV at the FLI by using the QIAmp Viral RNA Mini Kit (Qiagen, Hilden, DE) from 140 µL volume of each avian field sample (combined swab) or by using 100 µL volume of each swine sample (nasal swab) within the NucleoMaq® VET Kit (Macherey–Nagel), according to the manufacturer’s instructions.

### qRT-PCR and qPCR

To determine the relative IAV viral load, we quantified the viral RNA by RT-qPCR using the LightCycler Multiplex RNA Virus Master (Roche, Basel, CH) combined with previously described primers [20]. Briefly, 2 μL of RNA template was added to the reaction mixture composed of 1 × RT-qPCR Reaction Mix, 1 × RT Enzyme Solution, 0.8 μM of each forward (SVIP-MP-F) and reverse (SVIP-MP-R) primers, 0.2 μM of the probe (SVIP-MP_P2-MGB), supplemented to a total volume of 10 μL with PCR-grade water. The RT-qPCR analysis was performed on a LightCycler 480 (Roche) with an RT step for 10 min at 50 °C, followed by a heat-inactivation step for 30 s at 95 °C. The was followed by 45 cycles of denaturation (5 s, 95 °C) and annealing and elongation steps (30 s, 60 °C) with fluorescence readout. Finally, the cycling was concluded with a cooling step (30 s, 40 °C).

To assess the cDNA yield produced by various whole-genome sequencing protocols, we quantified 2 µL of A(H1N1)pdm09 cDNA generated using the Zhou protocols (2009 and 2012), the Rambo-Martin protocol (2020), and our optimized protocol via qPCR [11, 12, 15]. Quantification was performed using the Luna Universal Probe qPCR Master Mix (New England BioLabs Inc. (NEB), Ipswich, MA, USA) in combination with previously described primers, following the manufacturer’s guidelines [21]. Analysis was performed on a LightCycler 480 (Roche), where absolute copy numbers were calculated using a standard curve, followed by normalization to the amount of RNA template used during the RT stage of the respective protocols.

### Whole-genome amplification

For the optimized whole-genome amplification protocol, we used the LunaScript RT Master Mix Kit (Primer-free) (NEB) for the RT. For this, we used the previously described MBTuni-12 and MBTuni-12.4 primers in a ratio of 1:4 at a final molarity of 0.5 μM, and 7.5 μL of RNA eluate as input [15]. The cDNA synthesis consisted of two steps (2 min at 25 °C and 30 min at 55 °C) followed by heat-inactivation of the RT enzyme (1 min, 95 °C). Following the RT, we used 2.5 µL of cDNA as a template for a 25 µL PCR reaction with 0.02 U/μL of Q5 Hot Start High-Fidelity DNA Polymerase (NEB) and 200 μM dNTP mix (Promega, Madison, WI, USA), 0.5 μM of each primer MBTuni-13 (5′-ACG CGT GAT CAG TAG AAA CAA GG-3′) and MBTuni-12.4R (5′-ACG CGT GAT CAG CRA AAG CAG G-3′) or with barcoded primer pairs (Uni13-BCxx, Uni12-BCxx; Table S2). The PCR cycling protocol included an initial denaturation step of 30 s at 98 °C, followed by 35 cycles of denaturation (10 s at 98 °C), annealing (20 s at 64 °C), and elongation (105 s at 72 °C), concluding with a final elongation step of 5 min at 72 °C. The protocols from Zhou et al. using the SuperScript™III One-Step RT-PCR system (ThermoFisher) and Rambo-Martin et al. using SuperScript™IV Reverse Transcriptase (ThermoFisher) and Q5 DNA polymerase (NEB) enzymes were all performed as previously described [11, 12, 15].

### Library preparation and sequencing

Following PCR amplification, amplicons were subjected to a size selection using AMPure XP Bead-Based Reagent (Beckman Coulter, Brea, CA, USA) at a 0.5 × ratio to remove PCR amplicons smaller than 500 bp. After adding the beads to the PCR amplicons, the plate was loaded in the KingFisher Apex automated extraction instrument (ThermoFisher Scientific) using a custom in-house protocol. After the initial mixing and binding of the amplicons with the beads, two sequential washing steps with 80% ethanol were performed, followed by a bead drying step, and finished with the elution in the same volume of the input of nuclease-free water. For both the single- and dual-barcoding approaches, equal volumes of each amplified sample were used. For the dual-barcoding approach, samples amplified using barcoded primers were combined into pools of eight PCR-barcoded samples each. Each individual pool was quantified using the Qubit 1X dsDNA HS Assay Kit (ThermoFisher Scientific) on a Qubit 4 fluorometer (ThermoFisher Scientific). In the single-barcoding approach, no pooling was performed and samples were quantified using the same method to determine the maximum volume that could be loaded without overloading, based on the most concentrated samples. This volume was then applied uniformly across all single-barcoded samples for library preparation. For the ligation-based nanopore sequencing library preparation (SQK-NBD114.96, Oxford Nanopore Technologies (ONT), Oxford, UK), a maximum of 210 ng (200 fmol) was used as input according to the manufacturer’s instructions. The sequencing was done on a MinION Mk1B or GridION X5 device (ONT) on a MinION flow cell (R10.4.1, FLO-MIN114, ONT) and generated POD5 files were re-basecalled on the High Performance Computing (HPC) cluster of the University of Bern using the Dorado (v0.7.3, ONT) basecaller and the high-accuracy (hac) basecalling model (v4.3.0) from ONT [22].

### Data analysis

**Fig. 1:**
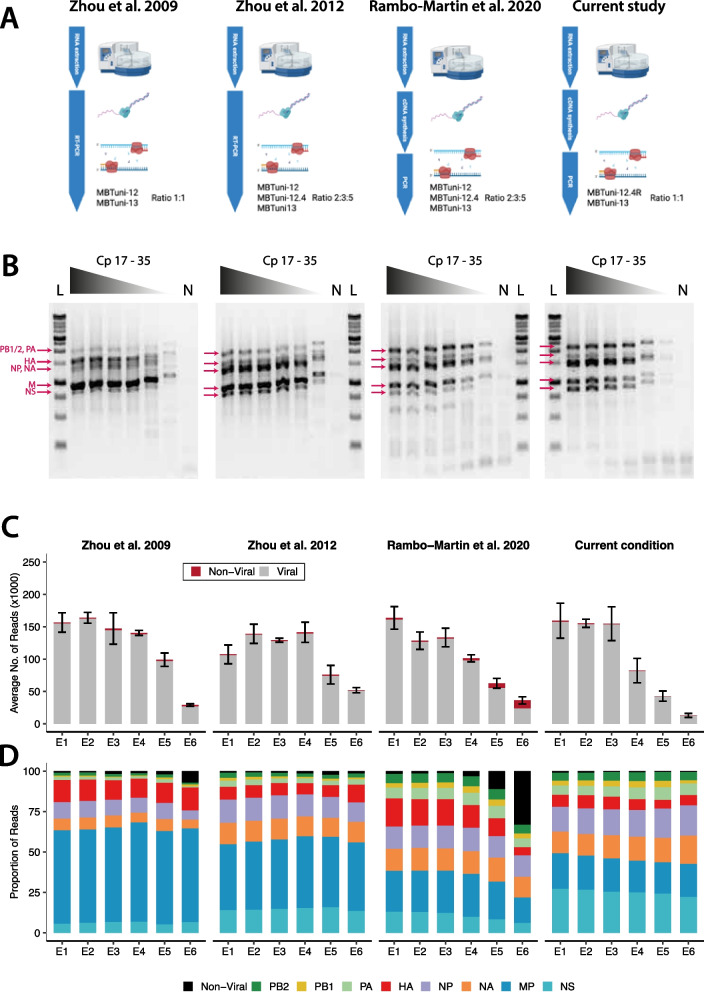
A modified two-step approach increases IAV whole-genome recovery. **A** Schematic overview of the different RT and PCR stages, and primer ratio used by the Zhou et al. [11, 12], Rambo-Martin et al. [15], and current optimized IAV WGS protocols (created in BioRender. Dijkman, R. (2025) https://BioRender.com/l62f505). **B** To compare the performance of the different IAV whole-genome amplification protocols, we used RNA extracted from six tenfold serial dilutions (Cp value range 17–35) of cell-cultured IAV supernatant as a template for the RT and subsequent PCR stages. The PCR products were resolved on an agarose gel where the individual amplicons corresponding to the genomic segments are annotated with an arrow (N = negative control; L = ladder). **C** PCR products from the tenfold serial dilution series for each protocol were sequenced to validate the agarose gel results. The resulting data are plotted as the average number of mapped viral (gray) and non-viral (red) reads per protocol. Results are presented as the mean ± standard deviation from three biological replicates. **D** To assess the recovery of all eight IAV genomic segments across protocols and dilutions, sequencing reads were subsampled for comparison (*n* = 8463 reads/sample to match the sample with the lowest number of sequencing reads). Viral segments are color-coded (with distinct hues) as PB2 (dark green), PB1 (yellow), PA (light green), HA (red), NA (orange), NP (purple), MP (teal), and NS (cyan), and non-viral sequences (black). Results are presented as the mean proportion of three biological replicates

**Fig. 2:**
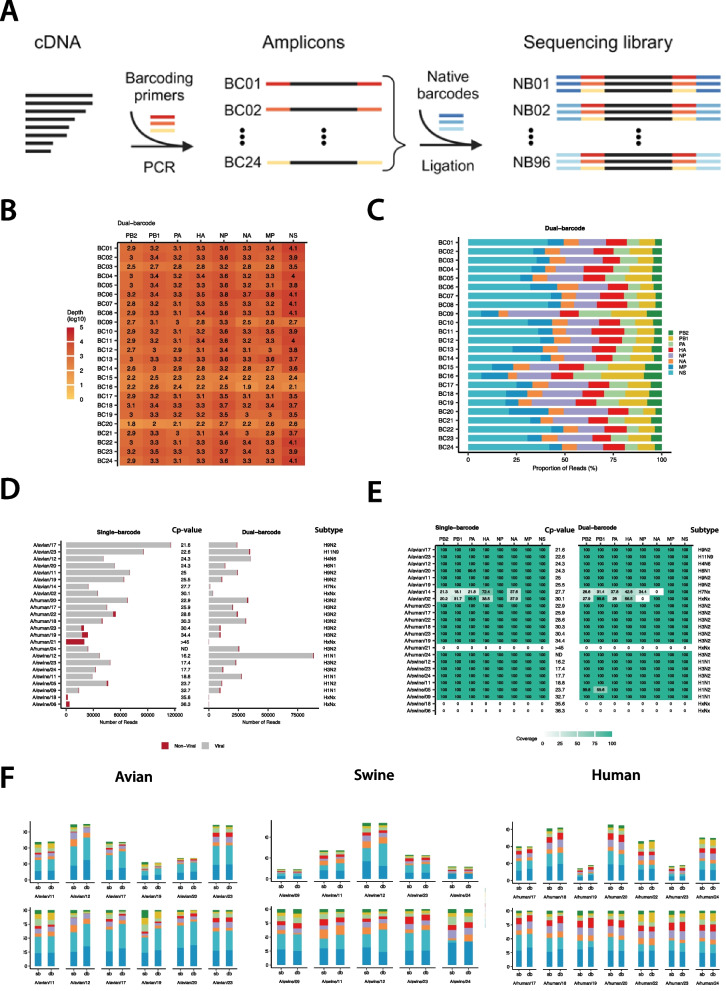
Dual-barcodes for high-throughput whole-genome sequencing. **A** Schematic overview of the dual-barcoding methodology. After amplifying the cDNA with unique PCR barcodes (red, orange, yellow), different samples are pooled and used as input for a single ONT native barcode from the sequencing library preparation kit (shades of blue). Finally, each amplicon will have two barcodes to identify the sample origin (created in BioRender. Dijkman, R. (2025) https://BioRender.com/f53z678). **B** To evaluate the whole-genome sequence recovery efficiency of the 24 custom PCR barcode primer sets, segment-level sequencing depth for each custom PCR barcode from dual-barcoded A(H1N1)pdm09 samples was plotted in a heatmap (full non-subsampled dataset). **C** Proportion of IAV genomic segments recovered for each custom PCR barcode from the dual-barcoded A(H1N1)pdm09 samples shown in **B**. Sequencing reads were subsampled for comparison (*n* = 1892 reads/sample to match the sample with the lowest number of sequencing reads). Viral segments are color-coded (with distinct hues) as PB2 (dark green), PB1 (yellow), PA (light green), HA (red), NA (orange), NP (purple), MP (teal), and NS (cyan). **D** To evaluate our single (left) and dual (right) barcoding strategies using the optimized protocol, we assessed the total number of viral (gray) and non-viral (red) sequencing reads recovered from 24 IAV-positive clinical samples from avian (A/Avian/XX), swine (A/Swine/XX), and human (A/Human/XX) origin. **E** The corresponding sequence coverage for each IAV genomic segment (shaded based on percentage) for the 24 clinical samples shown in **D** was plotted in a heatmap. **F** For pairwise comparison of the genomic recovery between single- and dual-barcoding strategies, samples from avian, swine, or human origin, where a complete genome could be recovered were subsampled based on the lowest number of total raw sequencing reads per clinical sample. The top bar graph shows the number of viral reads after subsampling for each sample, while the lower bar graph shows the proportion of reads mapped to IAV. Viral segments are color-coded (with distinct hues) as PB2 (dark green), PB1 (yellow), PA (light green), HA (red), NA (orange), NP (purple), MP (teal), and NS (cyan)

Before analysis, the output from the Dorado basecaller was demultiplexed into per-barcode Binary Alignment Map (BAM) files based on the native barcoding sequencing library kit used (SQK-NBD114.96), without trimming and requiring barcodes at both ends of each sequencing read (dorado demux command with –no-trim and –barcode-both-ends options enabled). For samples with native barcodes only, files were demultiplexed into individual Fastq files for each sample (single-barcode data in Figs. 1C, D, 2D–F, Additional file 1: Fig. S2A, B, Additional file 1: Fig. S4, Additional file 1: Fig. S5, and Table S2). For dual-barcoded samples, a second round of demultiplexing was performed after the first round of demultiplexing based on the native barcodes. To enable reclassification of reads in a subsequent round of demultiplexing, the barcode tag (BC) of each BAM file was first modified using Samtools (v1.13) [23] and modified BAM files were then demultiplexed again using a custom barcode kit arrangement based on our custom PCR barcodes (dorado demux command with the barcode-arrangement custom_dual_barcodes.toml –no-trim and –barcode-both-ends options enabled) [24]. Samples were emitted as individual Fastq files for each custom PCR barcode (dual-barcode data in Fig. 2B–F, Additional file 1: Fig. S3A–C, Additional file 1: Fig. S4, and Additional file 1: Fig. S5). For both single- and dual-barcode data, raw reads were filtered following demultiplexing using chopper (v0.9.2) to remove reads less than 500 bp and over 3000 bp in length [25]. To enable comparison across protocols, we normalized each dataset by downsampling the sequencing reads generated for each sample. For the dilution (Fig. 1D, Additional file 1: Fig. S2A, B) and dual-barcoded A(H1N1)pdm09 (Fig. 2C, Additional file 1: Fig. S3A, B) datasets, reads for each replicate were subsampled to match the filtered sequence read count of the replicate with the lowest number of reads in that dataset (dilution dataset *n* = 8463; dual-barcoded A(H1N1)pdm09 dataset *n* = 1892). For the clinical sample (Fig. 2F, Additional file 1: Fig. S4, Additional file 1: Fig. S5) dataset, reads were subsampled to match the filtered sequence read count of the protocol (single- or dual-barcode) with the lowest number of reads (Fig. 2F top panel; Table S5). The downsampling was performed by randomly sampling the reads using SeqKit (v2.8.0) tool kit (seqkit sample –n number_of_reads) [26]. This approach enabled optimal comparison of sequence read depth and coverage across dilutions and protocols.

For all datasets, complete genome assembly was performed using the FLU-minion module in Iterative Refinement Meta-Assembler (IRMA) software (v1.1.4) using default settings [27]. Estimates for sequence read depth of each barcoded sample were performed by re-mapping the reads to the generated consensus sequences using minimap2 software (v2.28) [28]. The resulting post-assembly metrics, including segment-specific sequence coverage and read depth, were summarized using in-house developed python scripts (available upon request). The data was further used for estimating average and standard deviation of viral and non-viral reads for each dilution. Data summaries were performed using dplyr (v1.1.4) package and visualized using ggplot2 (v3.5.1) in R v4.3.3 [29–31]. Computationally intensive tasks including basecalling, demultiplexing, read quality filtering, whole-genome assembly, and mappings were executed on UBELIX, the HPC cluster at the University of Bern.

## Results

### A modified two-step approach increases IAV whole-genome recovery and representation

The original multisegment RT-PCR (mRT-PCR) protocol developed by Zhou et al. [11], which targets the conserved nucleotide termini of each influenza A virus (IAV) genomic segment, along with subsequent modifications incorporating an additional primer, has been widely adopted for whole-genome surveillance of IAV at the human–animal interface [15, 16, 32–34]. Despite these advancements, recovering complete whole-genome sequence information remains challenging, particularly for samples with low viral loads. This holds true even for the two-step approach introduced by Rambo-Martin et al., which utilizes modified primer ratios to enhance sequencing coverage of the polymerase gene segments (i.e., PB1, PB2, and PA) [15]. We hypothesize that a key factor contributing to suboptimal whole-genome recovery of IAV is the use of suboptimal PCR reaction temperatures to facilitate primer binding at the conserved termini—an intentional feature of the original protocol (personal communication, Bin Zhou). We therefore aimed to investigate whether increasing the reaction temperatures during the reverse transcription (RT) and PCR stages could improve the dynamic range of complete viral gene segment recovery, particularly for low viral load samples. Specifically, we assessed the impact of elevated temperatures on full genome recovery and benchmarked our optimized workflow against the established whole-genome amplification protocols of Zhou et al. and Rambo-Martin et al. (Fig. 1A) [11, 12, 15]. For our optimized two-step workflow, we used the LunaScript Master Mix Kit and the Q5 DNA polymerase to minimize pipetting steps and reduce the need for additional auxiliary reagents (e.g., RNAse inhibitor) between the separate cDNA synthesis and PCR reactions [35]. Additionally, instead of using two independent MBTuni12 primers, as described by Zhou et al. and Rambo-Martin et al., we adopted a single degenerate Uni12 primer (MBTuni12.4R) to accommodate the U/C variation at the fourth nucleotide of the 3′ terminus (Fig. 1A) [12, 15]. This adjustment reduces handling complexity (i.e., primer ratio mixtures) while maintaining U/C variation segment coverage [12, 15]. Furthermore, we optimized the reverse transcription (RT) incubation temperature to 55 °C and applied a single annealing temperature of 64 °C during PCR, which was performed over 35 cycles. These temperature adjustments were designed to enhance cDNA synthesis and PCR amplification efficiency by aligning with the optimal reaction conditions of the enzymes used, thereby improving both yield and specificity [35].

To evaluate the impact of our modifications, we first tested six tenfold serial dilutions of a A(H1N1)pdm09 virus stock diluted in infection medium prior to RNA extraction and compared the performance of our workflow to the established IAV whole-genome amplification protocols described by Zhou et al. and Rambo-Martin et al. (Fig. 1A) [11, 12, 15]. As an initial step, we assessed whether there were differences in cDNA synthesis efficiency between the protocols. Directly quantifying cDNA is challenging, as it does not distinguish between IAV-specific and non-specific products. Therefore, we employed an M-segment-specific qPCR assay to assess the effectiveness of cDNA synthesis. This approach demonstrated that all protocols efficiently generated cDNA across all six tenfold dilutions (Additional file 1: Fig. S1). Notably, our modified protocol resulted in a slightly lower overall cDNA concentration for the M-segment compared to the established methods, while maintaining sufficient yield for downstream applications (Additional file 1: Fig. S1). Following the individual RT-PCR or two-step RT and PCR reactions, we resolved the amplified PCR products on an agarose gel (Fig. 1B). All amplicons corresponding to the eight genomic IAV segments were detectable across all methods and dilutions, with a concentration-dependent shift in band migration (Fig. 1B). Upon closer inspection, the Zhou et al. [12] protocol exhibited a more distinctive banding pattern at the lowest dilution compared to the parental Zhou et al. [11] protocol (Fig. 1B). Notably, both one-step amplification protocols [11, 12] showed disproportionate amplification of the HA and M segments (Fig. 1B). In contrast, the Rambo-Martin et al. [15] protocol displayed a less defined banding pattern at the lowest dilution and generally produced more amplicons of smaller size (Fig. 1B). By comparison, our modified protocol consistently yielded a clear and distinctive banding pattern across all dilutions, including the lowest, despite the slightly lower overall cDNA concentration of the M-segment, indicating an overall improved specificity and balanced amplification of all genomic segments (Fig. 1B, Additional file 1: Fig. S1).

To determine whether different protocols and input dilutions bias whole-genome segment recovery, we performed full-length amplicon sequencing using the nanopore sequencing platform. Prior to library preparation, we carried out a magnetic bead-based size selection step to remove smaller amplicons (≤ 500 bp) that were present at equal or higher molecular abundance than the target genomic segments, as these could distort the amplicon size distribution in the sequencing library. This enrichment for larger amplicons ensured a more accurate representation of complete IAV genome segments generated by the different whole-genome amplification protocols. Because an identical volume—rather than a molar-normalized input—was used for each protocol and dilution, the average number of sequencing reads per sample mirrored the expected patterns from the serial dilutions observed in the gel images (Fig. 1B and C). Sequencing analysis showed that the majority of reads across all protocols mapped to IAV (Table S3). Importantly, the proportion of viral reads was consistently higher for our optimized protocol, especially at lower input concentrations (Fig. 1C, Table S3). In contrast, the Rambo-Martin et al. [15] protocol yielded a substantially higher fraction of non-viral reads at low dilutions than all other protocols. This trend remained after normalization of sequencing read counts across protocols and dilutions (Fig. 1D). Furthermore, our protocol consistently achieved full genome recovery with relatively balanced read distribution in all eight genomic segments across all dilutions. In contrast, both Zhou et al. [11] and Zhou et al. [12] protocols showed a pronounced bias toward the M segment (Fig. 1D and Additional file 1: Fig. S2A). This bias may be attributed to a slightly higher abundance of M-segment-specific cDNA generated by these protocols compared to our approach (Additional file 1: Fig. S1). In addition to improved segment representation, our protocol also achieved a more uniform read depth along the full length of each segment (Additional file 1: Fig. S2B).

Together, these results demonstrate that our optimized protocol not only improves the representation and recovery of all eight IAV genome segments, but also ensures more consistent and even coverage, making it better suited for high-quality IAV whole-genome sequencing across a range of input concentrations.

### Scalable dual-barcoding approach enables high-throughput whole-genome sequencing of IAV

Although the portable third-generation sequencing platform from ONT is often used for the genomic surveillance of IAV, its throughput, namely the number of samples per run, is currently restricted by the number of available barcodes provided by the sequencing libraries (i.e., 24 or 96). Because our optimized protocol increases IAV whole-genome recovery, and since Rambo-Martin et al. used barcoded primers for their sequence library preparation after the whole-genome amplification stage, we were inspired to evaluate whether it would be possible to generate a dual-barcoding strategy for a scalable high-throughput approach for IAV whole-genome sequencing [15].

To enable multiplexed amplification and sequencing, we designed custom barcoded primer sets for the PCR amplification step, replacing the previously used MBTuni12R and MBTuni13 primers that target the conserved 3′ and 5′ termini of the eight IAV genomic segments. The barcoded primer sets incorporated the first 24 barcode sequences from the ONT PCR Barcoding Expansion Kit (EXP-PBC096), each flanked by additional sequences to facilitate accurate demultiplexing (Fig. 2A, Table S4). The resulting design is compatible with the commercially available ONT Native Barcoding Kits SQK-NBD114.24 and SQK-NBD114.96. To validate the performance of the 24 custom PCR barcodes for IAV whole-genome recovery, we generated cDNA from extracted RNA from a A(H1N1)pdm09 virus stock using our optimized two-step amplification protocol. This cDNA was then used as a template in parallel reactions with each of the 24 custom PCR barcode sets. Following amplification, amplicons from eight individual barcoded primer reactions were pooled in equal volumes and barcoded using the ONT Native Barcoding Kit before sequencing. This revealed that irrespective of the custom PCR barcode used, all eight genomic segments of the IAV genome could be recovered with at least 50 × depth per segment (Fig. 2B, Table S4). Notably, four custom PCR-barcoded primer sets showed slightly reduced sequence recovery for certain genomic segments (BC09, BC15, BC16, and BC20) (Fig. 2B). This trend remained evident after normalization of sequencing read counts for each barcoded primer set (Fig. 2C, Additional file 1: Fig. S3A, Additional file 1: Fig. S3B), likely due to an overall lower number of reads associated with these barcodes (Fig. 2B, Additional file 1: Fig. S3C, Table S4). However, enough sequence reads were still generated to recover the full-length sequences of all eight genomic segments (Fig. 2C). This demonstrates that a dual-barcoding approach can be effectively applied to whole-genome sequencing of IAV, offering the potential to increase library preparation throughput by at least eightfold. Notably, downsampling analyses showed that our protocol maintains high segment recovery even at reduced read depths, underscoring its suitability for samples with low viral loads.

### High-throughput whole-genome sequencing of IAV from diverse clinical samples

To evaluate the applicability of our optimized two-step approach—including both single- and dual-barcoding strategies—for whole-genome sequencing of IAV from clinical samples, we tested 24 IAV-positive specimens derived from human, swine, and avian hosts (eight per host). These samples represented a broad range of Cp values (16–36) and multiple IAV subtypes, including avian (H4N6, H5N1, H6N1, H7N7, H9N9, and H11N9), human (H3N2), and swine (H1avN2, H1pdmN1, H1huN2, and H3huN2) viruses. Following RNA extraction and reverse transcription, IAV genomes were amplified using the previously customized MBTuni (single-barcoded samples) or PCR-barcoded primer sets (Table S2). For the latter, amplicons from eight individually PCR-barcoded samples were pooled in equal volumes and then barcoded using the ONT native barcoding kit (dual-barcoded samples). Following sequencing, we assessed the overall sequencing output for each approach, including the proportion of viral versus non-viral reads, sequencing depth and coverage, and genomic segment Distribution before and after normalization of sequence read counts per sample. The optimized protocol successfully recovered viral reads from 21 of the 24 samples, irrespective of barcoding strategy (Fig. 2D). However, in three samples (A/Human/21, A/Swine/18, and A/Swine/06), no viral reads were recovered, and thus, no consensus sequences could be generated, possibly due to the low viral load in these samples (Fig. 2D and E and Table S1; Cp value). For sample A/Swine/05, a complete full genome was recovered using the single-barcoding strategy, while only a near-complete full genome was recovered for the dual-barcoding strategy, as the PB1 segment was only partially covered (59%) (Fig. 2E). Only the smaller IAV genomic segments were recovered from two avian samples (A/Avian/02, A/Avian/14), which had Cp values of 27.7 or higher, from which A/Avian/02 could only be partially subtyped in the single-barcoding strategy (Fig. 2E). These results likely reflect the low number of sequencing reads acquired for each pool of samples from the dual-barcoding strategy—only ~ 160,000 per native barcode—compared to the single-barcoding strategy (Fig. 2D, Table S5). Except for A/Human/21, A/Swine/18, and A/Swine/06, the proportion of non-viral reads between both barcoding strategies did not differ substantially (Fig. 2D, Additional file 1: Fig. S4).

To compare the Distribution of reads across gene segments between the single- and dual-barcoding approaches, we normalized read counts per sample to the lowest read count observed across both protocols. This comparison was applied only to samples where full genomes were recovered using both approaches. The Distribution of reads for recovered gene segments among the 18 whole genomes (6 for avian, 5 for swine, and 7 for human) was largely comparable for the human and swine samples (Fig. 2F). For the avian IAV-positive samples, two of the six samples displayed a similar distribution of gene segment recovery, while the remaining four exhibited more variable recovery patterns. Nonetheless, both the single- and dual-barcoding approaches achieved sufficient depth to generate consensus sequences for each genomic segment and enable sub-typing of these samples (Fig. 2D and E).

Third-generation sequencing platforms, which enable full-length sequencing of individual DNA amplicons, make it theoretically possible to detect defective viral genomes (DVGs) generated during IAV infection. We therefore evaluated whether DVGs could be detected in our set of IAV-positive clinical samples of human, swine, and avian origin. For this, we employed a splice-aware aligner and assembled the 5′ and 3′ termini of possible DVGs, as done previously for influenza D virus, and then determined the sequencing coverage across each IAV genomic segment [36]. Consistent with the presence of DVGs, this revealed that several samples exhibited a sharp drop in coverage across the middle region, especially in the PB1, PB2, and PA segments, whereas such patterns were absent from other samples (Additional file 1: Fig. S5). Although the underlying reason for the presence/absence of DVGs in certain samples remains unclear, our analysis indicates that our approach can readily detect DVGs in clinical samples from avian, swine, and human origin. Combined, these results demonstrate that with our revised two-step PCR protocol, both the single- and dual-barcoding strategies can be used for WGS of IAV-positive samples originating from diverse host species using a portable third-generation sequencing platform.

## Discussion

In the present study, we demonstrate that modifying the RT and PCR cycling conditions significantly enhances the representation and recovery of all eight IAV genome segments, achieving consistent, even coverage across a wide range of viral loads compared to existing protocols. Additionally, incorporating custom PCR-barcoded primers enables multiplexing of at least eight IAV-positive clinical samples—spanning avian, swine, and human origins—per ONT sequencing library without compromising IAV genome recovery. Together, these improvements establish a streamlined and scalable workflow for high-throughput whole-genome surveillance of influenza A virus across diverse host species.

Since the description of the whole-genome RT-PCR approach by Hoffmann et al. in 2001 [37], using individual segment-specific primers, and the subsequent development of a single-primer set approach by Zhou et al. in 2009 [11], numerous adaptations and modifications of these methods have been reported by various research groups tailored for use with the ONT sequencing platform [15, 16, 33, 34]. However, to our knowledge, this is the first systematic benchmark of a newly optimized IAV whole-genome amplification protocol against earlier single-primer set methods using cell culture samples, with additional validation on clinical samples. While clinical samples were not benchmarked against using previous methods, successful full genome recovery from diverse clinical samples supports our protocol’s suitability for use in diagnostic and surveillance settings. Our optimized two-step protocol consistently produced a higher proportion of viral reads and more uniform genome coverage, even at low RNA input concentrations. Specifically, ≥ 98% of demultiplexed ONT reads were influenza-specific, outperforming previous reports such as King et al., who reported 91.3% of all influenza reads [33]. This high specificity, coupled with the ability to recover full genomes from a wide variety of IAV-positive clinical samples from avian, swine, and human origin, highlights the critical importance of modifying the RT and PCR cycling conditions to enhance amplification efficiency and reduce non-specific background. These improvements were evident with both barcoding strategies, with the dual-barcoding approach enabling high-throughput multiplexing of IAV-positive samples. Together, these findings underscore the robustness and scalability of our workflow for whole-genome sequencing of IAV across diverse host species and viral loads.

Here, we demonstrate that dual-barcoding is possible on the ONT sequencing platform with up to eight samples per native ONT sequencing barcode library. Based on the number of barcodes available for the ONT PCR barcoding expansion kit (EXP-PBC096), this can, in principle, be expanded to 96 samples per native ONT sequencing barcode library. However, in our experimental settings, we Did not quantify individual barcoded samples, but only the pool of samples to ensure that each native ONT sequencing barcode Library was made with 200 fmol. Therefore, including more barcodes could dilute out samples from which the amplicon concentration is low. To overcome this, one needs to quantify individual barcoded samples, normalize them during pooling, and empirically determine the maximum number of individual barcoded samples that can be pooled per native ONT sequencing barcode library. This likely will further reduce the number of native ONT sequencing barcode libraries needed, without compromising full genome recovery detection sensitivity, but would be more laborious compared to the “blinded” pooling approach described in the current study, and alternatively can be resolved with a deeper sequencing depth. Finally, although the dual-barcoding approach described here has thus far only been evaluated for WGS, it could readily be adapted to other multisegment and multiplex approaches for genomic surveillance of seasonal human influenza A and B viruses [38, 39].

The multisegment RT-PCR is an essential tool for performing whole-genome sequencing of IAVs. We show that our optimized protocol can be used to sequence IAV-positive samples from avian, swine, and human origin up to a Cp value of approximately 34. However, we observed that samples of avian origin had, in general, a reduced genomic recovery compared to the samples of swine or human origin, which had even lower viral loads than the avian IAV samples (Fig. 2C). This discrepancy suggests that sample origin can significantly influence detection sensitivity. However, we cannot exclude that this might be influenced by the fact that the avian and swine samples, in contrast to the human samples, underwent several freeze/thaw cycles prior to the experiments, which can negatively affect RNA quality and integrity. Similarly, the avian samples were extracted manually with a column-based kit, whereas the human and swine samples were extracted using an automated magnetic bead-based method, resulting in the avian samples having a lower sequencing sensitivity, which aligns with a previous report [40]. Given that IAVs cause acute infections, the time of sampling relative to the time of infection is important, as viral loads in clinical samples decrease toward the end of illness [41, 42]. Since we did not recover a complete full genome for all IAV-positive clinical samples, a more controlled study would be valuable to assess whether full genome recovery rates are influenced by sample origin, sample processing, and time of sampling.

Because the exact role of defective viral genomes (DVGs) in vivo and in vitro during virus infection remains largely elusive [43], several short-read-based bioinformatic pipelines have been established to analyze this phenomenon [44, 45]. Thus, while detecting DVGs for IAV is not novel per se, we demonstrate that in addition to recovering whole-genome information, our WGS approach can also be used to detect IAV DVGs in clinical specimens, without any potential fragment partitioning during sequence library preparation [45]. However, because DVGs of PB1, PB2, and PA are on average 400–500 nt in size, and HA, NA, NP, M, and NS DVGs are on average around 400 nt, it is possible that the size selection step of our protocol prior to sequencing depletes some of the DVG amplicons shorter than 500 bp. This likely reflects why most DVGs we detected originated from the largest genomic segments of IAV (e.g., PB1, PB2, and PA) (Fig. S2). Therefore, it remains to be determined if our approach can reliably detect DVGs of the small genomic segments of IAV, as well as how long-read NGS methodologies compare to short-read NGS approaches.

## Conclusions

In conclusion, our optimized and scalable whole-genome sequencing workflow for influenza A virus—enhanced through modified RT and PCR cycling conditions and dual-barcoding—enables high-throughput sequencing of IAV-positive samples from avian, swine, and human origins with consistent genome recovery, even at low viral loads. It provides a practical and robust solution for IAV genomic surveillance at the human–animal interface, supporting early detection, monitoring virus evolution, and rapid identification of reverse zoonotic and zoonotic spillover events.

## Supplementary Information

Additional file 1: Supplementary figures 1–5.

Additional file 2: Supplementary Table 1 Metadata of RNA samples extracted from IAV-positive clinical samples or virus isolates of avian, swine, and human origin.

Additional file 3: Supplementary Table 2 Barcoded primers table. The table includes all the barcoded primers designed and used in this study. The underlined nucleotides at the end of each primer sequence correspond to the conserved nucleotides at each IAV segment end (i.e., Uni12 and Uni13).

Additional file 4: Supplementary Table 3 Sequencing metrics summary underlying Fig. 1C. This table provides global sequencing read statistics and classification of viral and non-viral reads for serially diluted samples amplified using the protocols of Zhou et al. [11, 12], Rambo-Martin et al. [15], and the current optimized protocol.

Additional file 5: Supplementary Table 4 Sequencing metrics summary underlying Fig. 2B. This table provides global sequencing read statistics, the number of reads classified as viral and non-viral, and reads mapped per genomic segment with the dual-barcoding approach.

Additional file 6: Supplementary Table 5 Sequencing metrics summary underlying Fig. 2D. This table provides global sequencing read statistics and the number of reads classified as viral and non-viral for each IAV-positive clinical sample sequenced with either the single- or dual-barcoding approach.

## Data Availability

Data generated in this study have been deposited in the European Nucleotide Archive (ENA) under the accession number PRJEB79943 (https://www.ebi.ac.uk/ena/browser/view/PRJEB79943). All clinical samples - regardless of avian, swine, or human origin - were processed uniformly, with human-identifiable reads removed, where applicable, prior to submission to the ENA (Table S5). Barcode arrangement file, scripts for figure generation, and baseline data underlying this work are made available at Zenodo (https://zenodo.org/records/15774804) [46].
