# Supplementary material for: Optimized high-throughput whole-genome sequencing workflow for surveillance of influenza A virus
Source: Genome Med. 2025 Sep 26;17:103. doi: 10.1186/s13073-025-01512-x (PMC12465963; doi:10.1186/s13073-025-01512-x)
Supplement: Supplementary file 1 — Additional file 1: Supplementary figures 1–5. [file 13073_2025_1512_MOESM1_ESM.pdf]

Fig S1

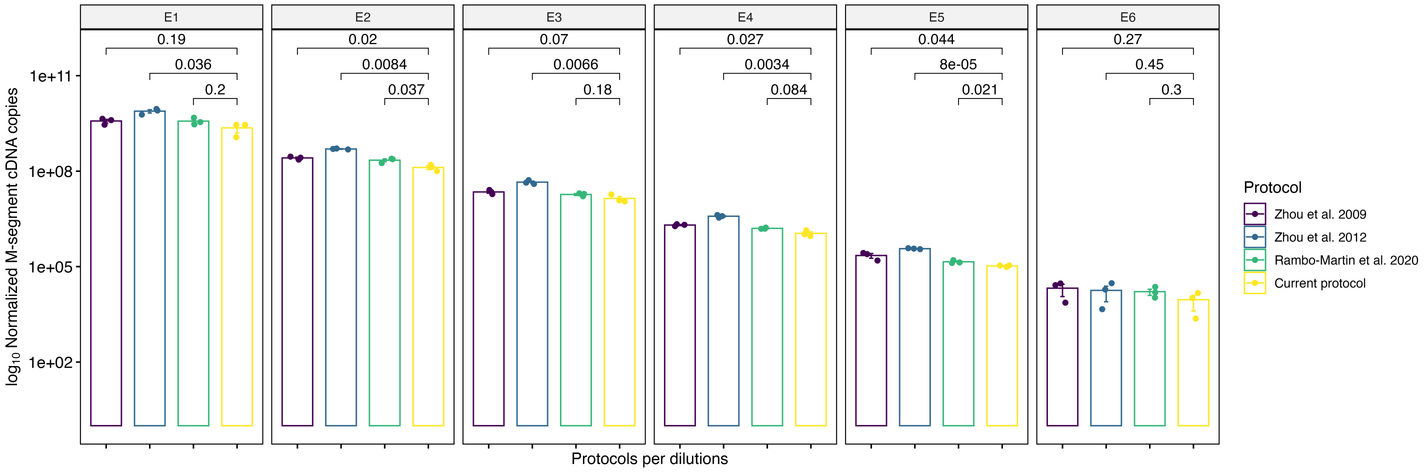

Quantification of M gene cDNA from different IAV whole genome sequencing protocols. Absolute cDNA copy numbers were measured by qPCR using 2 µL of A(H1N1)pdm09 cDNA synthesized via the Zhou (2009 and 2012), Rambo-Martin (2020), and optimized protocols. RNA input consisted of six 10-fold serial dilutions (Cp range 17–35). Quantification was performed using the Luna Universal Probe qPCR Master Mix and previously described primers. Absolute values were calculated using a standard curve and normalized to the amount of RNA template used during reverse transcription. Results are shown as mean ± standard deviation from three biological replicates. Statistical comparisons between selected protocols within each dilution were performed using two-sided t-tests; only significant p-values are displayed ($p < 0.05$).

Fig S2

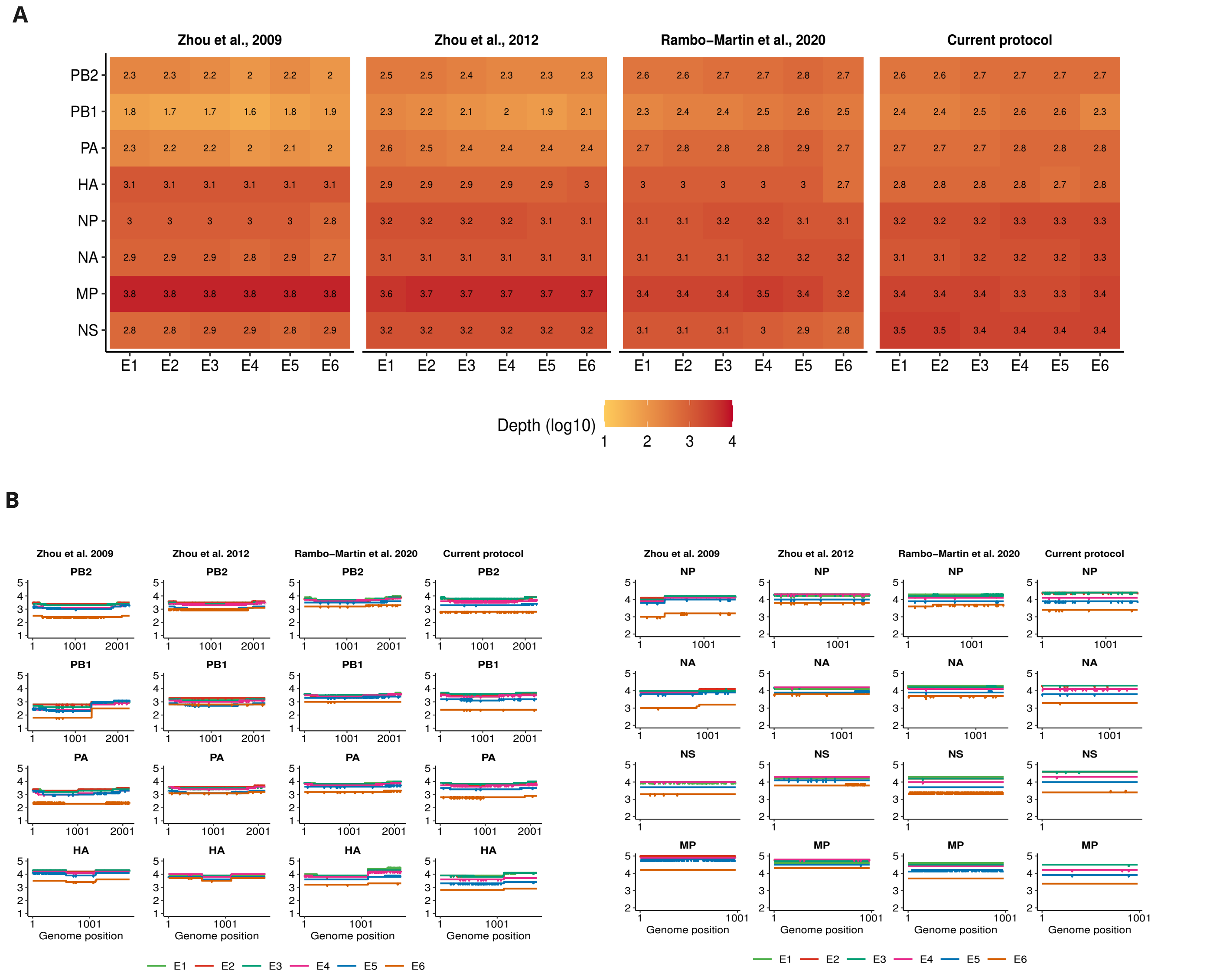

Normalized read depth across influenza A virus segments for different WGS protocols and RNA input dilutions. Mean read depth from three biological replicates, based on subsampled reads (n=1,892 reads/sample to match the sample with the lowest number of sequencing reads), is plotted as A) a heatmap illustrating segment-level sequencing depth and B) the number of sequencing reads (y-axis) across nucleotide positions (x-axis) for each IAV genome segment. Data are shown for the Zhou (2009 and 2012), Rambo-Martin (2020), and optimized protocols, across six 10-fold serial dilutions (Cp range 17–35). Different input dilutions are color-coded.

Fig S3

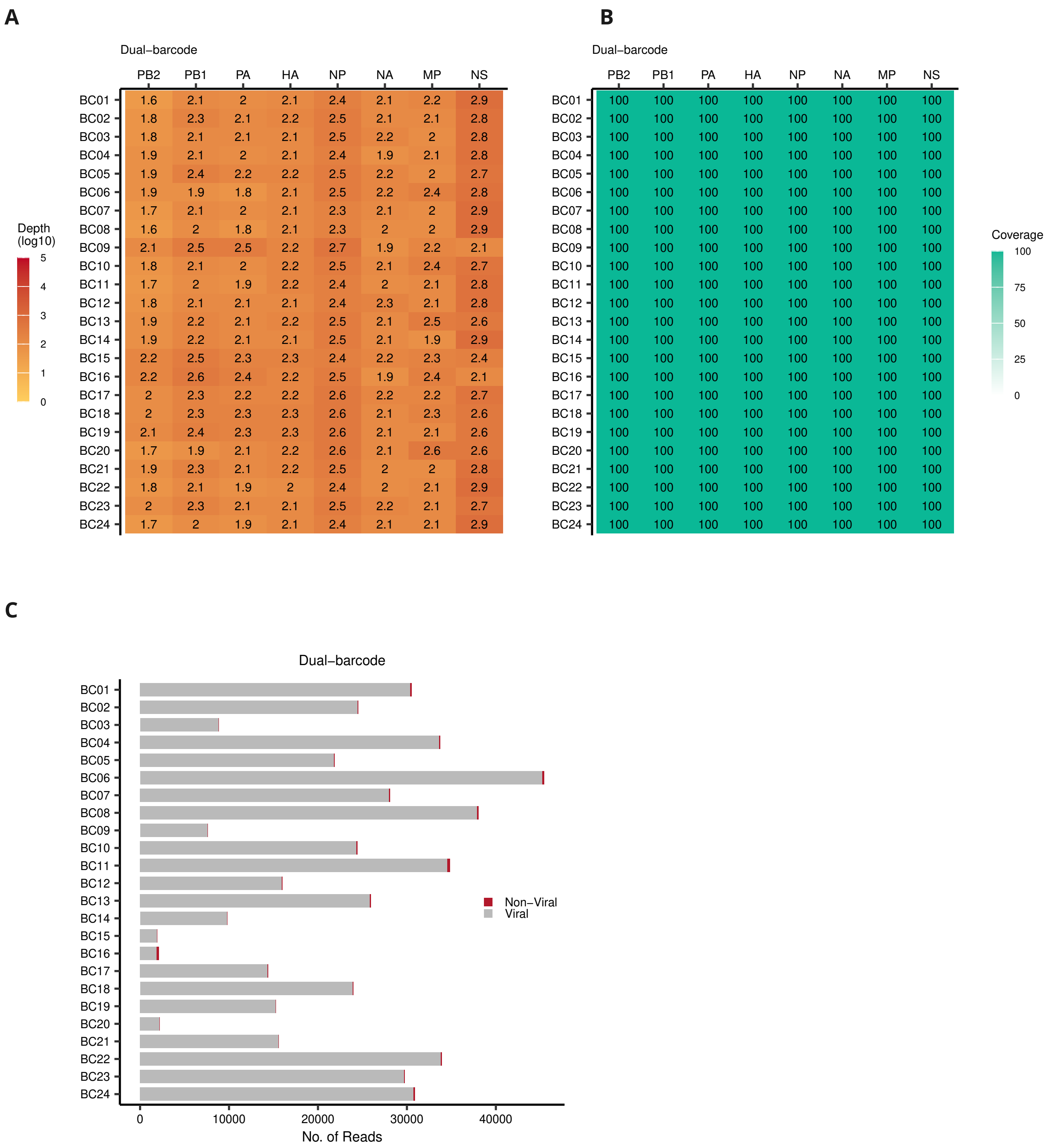

Segment-level read depth and coverage from dual-barcoded A(H1N1)pdm09 samples. Heatmaps display the number of reads mapped to each influenza A virus segment A) and the extent of segment coverage across nucleotide positions B), based on dual barcoding. Read depth was normalized across samples using subsampling to enable comparison of coverage breadth and sequence read distribution between the different PCR barcodes. C) Total number of viral (grey) and non-viral (red) sequencing reads from dual-barcoded A(H1N1)pdm09 samples (full non-subsampled dataset).

Fig S4

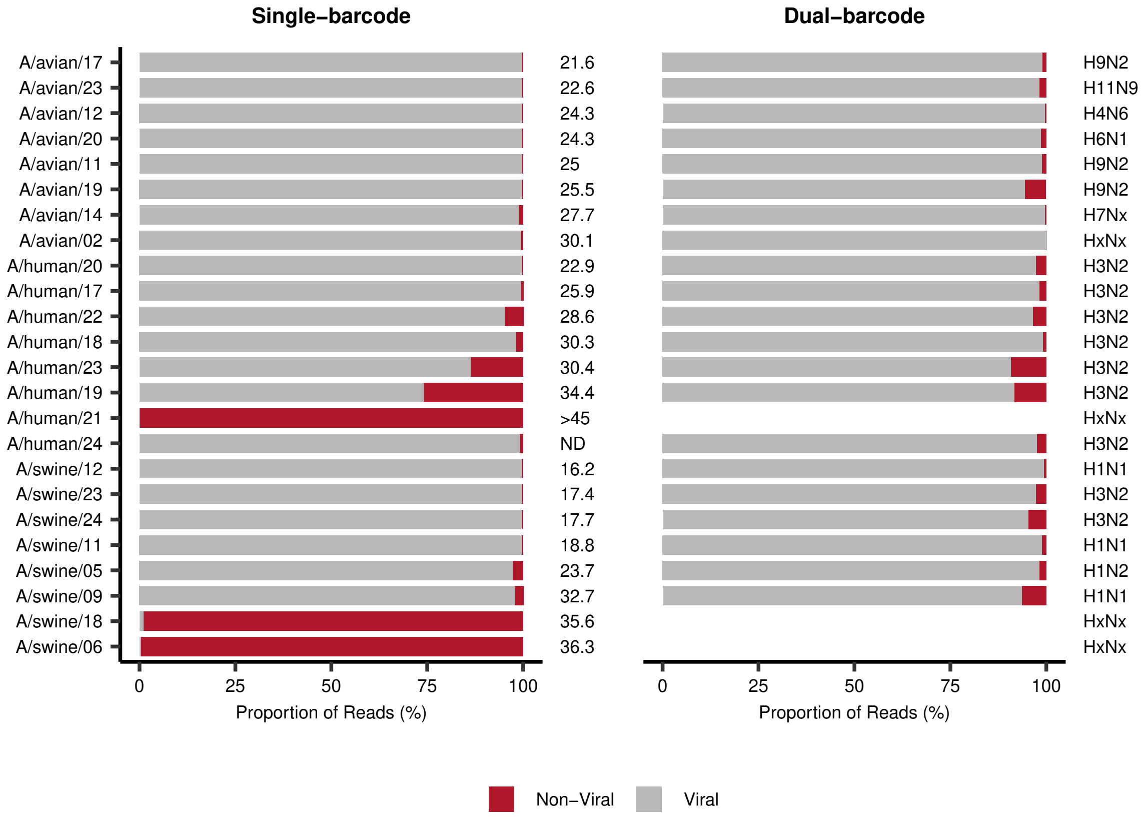

Proportion of viral and non-viral reads in sequence data from IAV-positive clinical samples. The proportion of mapped viral (grey) and non-viral (red) reads detected from IAV-positive clinical samples of avian, swine, and avian origin sequenced with a single or dual barcoding strategy.

Fig S5

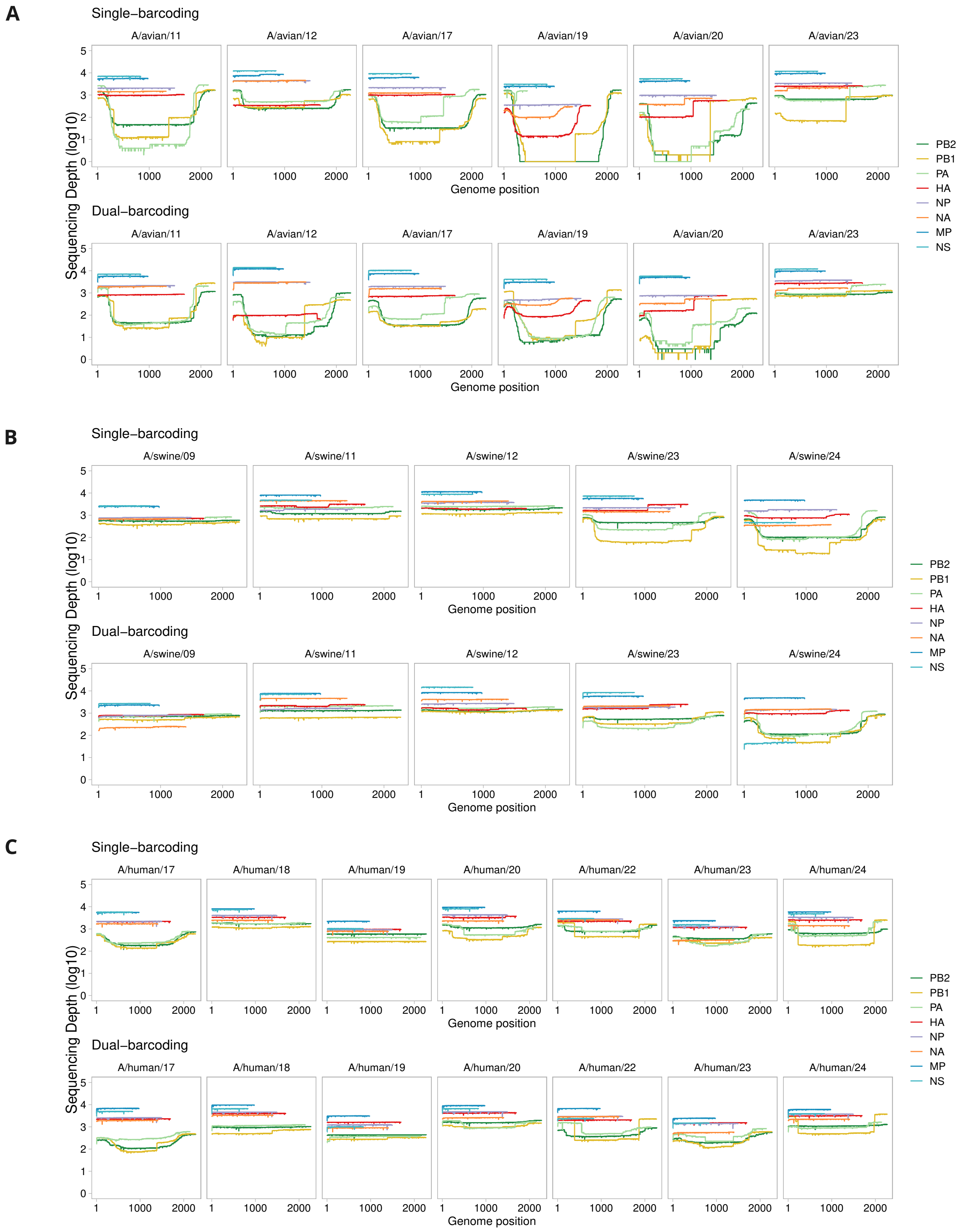

Normalized read depth across influenza A virus segments from IAV-positive clinical samples of avian, swine, and human origin. The read depth is plotted as the number of sequencing reads (y-axis) across nucleotide positions (x-axis) for each IAV genome segment. IAV-positive RNA samples from avian, swine, and human hosts were sequenced with the optimized WGS protocol using a single- or dual-barcoding strategy.
